# Assessment and influencing factors analysis of economic system vulnerability of the Belt and Road Initiative countries

**DOI:** 10.1371/journal.pone.0262611

**Published:** 2022-01-14

**Authors:** Zhihui Li, Jia Wu, Xiaolin Cui, Zhaojuan Mi, Lu Peng

**Affiliations:** 1 Key Laboratory of Land Surface Pattern and Simulation, Institute of Geographic Sciences and Natural Resources Research, Chinese Academy of Sciences, Beijing, China; 2 China-Pakistan Joint Research Center on Earth Sciences, CAS-HEC, Islamabad, Pakistan; 3 University of Chinese Academy of Sciences, Beijing, China; 4 College of Geomatics, Xi’an University of Science and Technology, Xi’an, Shaanxi, China; Northeastern University (Shenyang China), CHINA

## Abstract

Economic vulnerability is an important indicator to measure regional coordination, health and stability. Despite the importance of vulnerabilities, this is the first study that presents 26 indicators selected from the dimensions of the domestic economic system, external economic system and financial system in the Belt and Road Initiative (BRI) countries. A quantitative analysis is conducted to analyze the characteristics of spatial heterogeneity of vulnerability of the economic subsystems and the comprehensive economic system of the BRI countries and the main influencing factors of the comprehensive economic system vulnerability (CESV) are identified based on obstacle degree model. The results show that the CESV of the East Asia, South Asia and ASEAN countries are lower than that of the Middle Eastern Europe, Central Asia and West Asia countries. The CESV of the BRI countries are generally in the middle level and the average vulnerability index of highly vulnerable countries is twice as much as that of lowly vulnerable countries. In addition, in terms of the vulnerability of the three subsystems, the spatial distribution of vulnerability of the domestic economic system (DESV) and financial system (FSV) is basically consistent with the spatial distribution pattern of CESV, both of which are low in East Asia and South Asia and high in West Asia and Central Asia. While, the vulnerability of external economic system (EESV) shows a different spatial pattern, with vulnerability of West Asia, Central Asia and ASEAN higher than that of East Asia and South Asia. The main obstacle factors influencing the CESV of BRI countries include GDP growth rate, saving ratio, ratio of bank capital to assets, service industry level, industrialization level and loan rate. Therefore, the key way to maintain the stability and mitigate the vulnerability of the economic system of BRI countries is to focus on the macroeconomic development and operation, stimulate the economy and market vitality, promote the development of industries, especially the service and secondary industries, and optimize the economic structure, banking system and financial system.

## 1. Introduction

The Belt and Road Initiative (BRI) refers to the major strategic concept of “the Silk Road Economic Belt” and “the 21st Century Maritime Silk Road” [1]. The BRI focuses on policy connectivity, infrastructure connectivity, trade connectivity, financial connectivity and people-to-people connectivity. It also includes a cooperative framework of “six economic corridors, six road networks, several pivot countries and several marine pivot ports”, providing new ideas and solutions for improving the reform of the global governance system. According to the Belt and Road Portal of China, China has signed cooperation documents on the Belt and Road co-construction with 140 countries and 32 international organizations by June 2021 [2]. In 2018, China’s investment in the BRI countries reached USD 15.64 billion, with an increase of 8.9% compared to that in 2017, accounting for 12% of the total non-financial investment in foreign countries [1]. The investments were mainly in the manufacturing, mining, leasing and business services, electric and heat power supply, finance, infrastructure construction and other industries. In addition, the BRI countries have become China’s priorities of investment, and China’s “Going Out” strategy reached a new height. However, due to the large geographical span along the BRI region, most of the countries are developing countries and emerging economies and differ in natural environment and resources [3]. Also, these countries differ in political systems, economic development level, industrialization and urbanization processes, and ethnic and cultures and religions [4, 5]. These countries also have problems such as natural disasters, gaps between rich and poor and financial crisis etc., thereby leading to varying degrees of economic vulnerability in these countries. In recent years, vulnerability studies have attracted the attention of many scholars in the identification and solution framework of social and economic problems [6]. Therefore, understanding the comprehensive economic system vulnerability (CESV) of the BRI countries is crucial to promoting economic policy coordination of BRI countries, carrying out broader, higher-level and deeper regional cooperation, and jointly building an open, inclusive, balanced and universally beneficial regional economic cooperation architecture.

The concept of vulnerability was introduced into the field of natural disasters in the 1970s [7]. It was first proposed by Peter Timmerman in 1981 and has been widely used in multiple disciplines such as ecology, economics, sociology and geography [8–13]. With the rise of studies on global environmental changes, vulnerability studies have gradually become a frontier field in the studies of global environmental changes and sustainable science, and has also attracted the attention of several international scientific research programs and institutions, such as IHDP, IPCC and IGBP [14]. The concept of vulnerability was first considered as the possibility of damage to a system due to adverse effects such as disasters. Then, it has evolved into a concept set including “sensitivity”, “adaptability”, “resilience” and other elements with the deepening and improvement of its concept, evaluation method, analysis framework and research content. In addition, vulnerability studies have expanded from natural science to humanities and social sciences. For example, studies on early ecological vulnerability and environmental vulnerability developed into studies on economic vulnerability, urban vulnerability, livelihood vulnerability and others. Economic vulnerability is an important component of the concept and connotation of vulnerability. As an important measure of the developmental health and stability of a regional or national economic system. It also plays a significant role in economic and structural transformation as well as regional sustainable development. Briguglio proposed the economic vulnerability index, and first applied it to economic analysis in 1992 [15]. Since the United Nations Development Program (UNDP) formally put forward the concept of “economic vulnerability” in 1999 [16], researchers have carried out multi-level and multi-angle studies on economic vulnerability. Ria et al. established an economic vulnerability evaluation indicator system based on panel data of 23 countries, and put forward countermeasures to reduce economic vulnerability in the context of emerging market economies [17]. Aroca et al. collected 118 economic variables and constructed a new economic vulnerability index using factor scores calculated by the least square method, then analyzed and evaluated the economic vulnerability of urban areas prone to torrential floods by comprehensive assessment method [18]. Furthermore, Gnangnon explored the economic vulnerability of 112 developing countries under the liberalization of the multilateral trade policy [19]. Ren et al. comprehensively evaluated the provincial economic vulnerability and analyzed the spatial differences during economic growth by entropy, multilevel extensive evaluation and spatial difference analysis in the theoretical framework of vulnerability [20]. Also, Tang established a research framework of economic vulnerability of export-oriented cities, and analyzed the economic vulnerability of Foshan as the spatial and temporal evolution characteristic by “sensitivity-response” model, set-pair analysis, GIS spatial analysis and other methods [21]. In conclusion, studies generally evaluate the degree of economic vulnerability using quantitative methods, analyze the main influencing factors of economic vulnerability by constructing the CESV assessment model, and propose related policies and measures, and pay attention to theoretical explanation and systematic analysis. In terms of evaluation methods of economic vulnerability, scholars usually use such methods as principal component analysis (PCA), pressure-state-response (PSR) method, entropy weight methods, analytical hierarchy process (AHP) and fuzzy comprehensive evaluation etc. [20, 22, 23].

There are many problems existing in the BRI countries, such as unbalanced economic development among countries, low levels of foreign trade and imperfect infrastructure constructions. To assess the social and economic development along the Belt and Road, Zhang et al. constructed a comprehensive evaluation indicator system of the resilience of the BRI countries with 24 specific indicators. These indicators were selected from three dimensions by entropy and multi-index comprehensive evaluation, to evaluate the comprehensive resilience and spatial heterogeneity of China and other 64 BRI countries [24]. Ba et al. analyzed the adaptability, ecological vulnerability and resilience of the basin in BRI, focused on three basic dimensions of sustainable development, namely economic, ecological and social factors, and constructed a distribution map of the social and ecological resilience along the Belt and Road [25]. Furthermore, Li assessed the social and economic vulnerability of the BRI countries influenced by natural disasters, using the data envelopment analysis model and investigated the convergence characteristic of social and economic vulnerability among countries by Theil index [3]. Indicators selected and evaluation systems established for the assessment of social and economic development vulnerability along the Belt and Road are mostly inconsistent among scholars. Most focus on the assessment and ranking of vulnerability at a single time point, and few focused on the vulnerability of the system, from the dynamic change of evaluation indicators. Therefore, we construct an evaluation indicator system of CESV that include the vulnerability of the domestic economic system (DESV), the vulnerability of external economic system (EESV) and vulnerability of financial system (FSV) to comprehensively analyze the CESV of the 65 BRI countries in this study. According to the IPCC concept of vulnerability, the vulnerability of economic system is calculated according to sensitivity and adaptability based on the panel data of year 2000–2019 [26], and the obstacle factors influencing CESV based on the obstacle degree model are further identified. To reveal the spatial heterogeneity characteristic and key influencing factors of the comprehensive CESV of the BRI countries will provide a reference for reducing the economic vulnerability and achieving sustainable economic development along the Belt and Road.

## 2. Materials and methods

### 2.1 Study area

**Fig 1:**
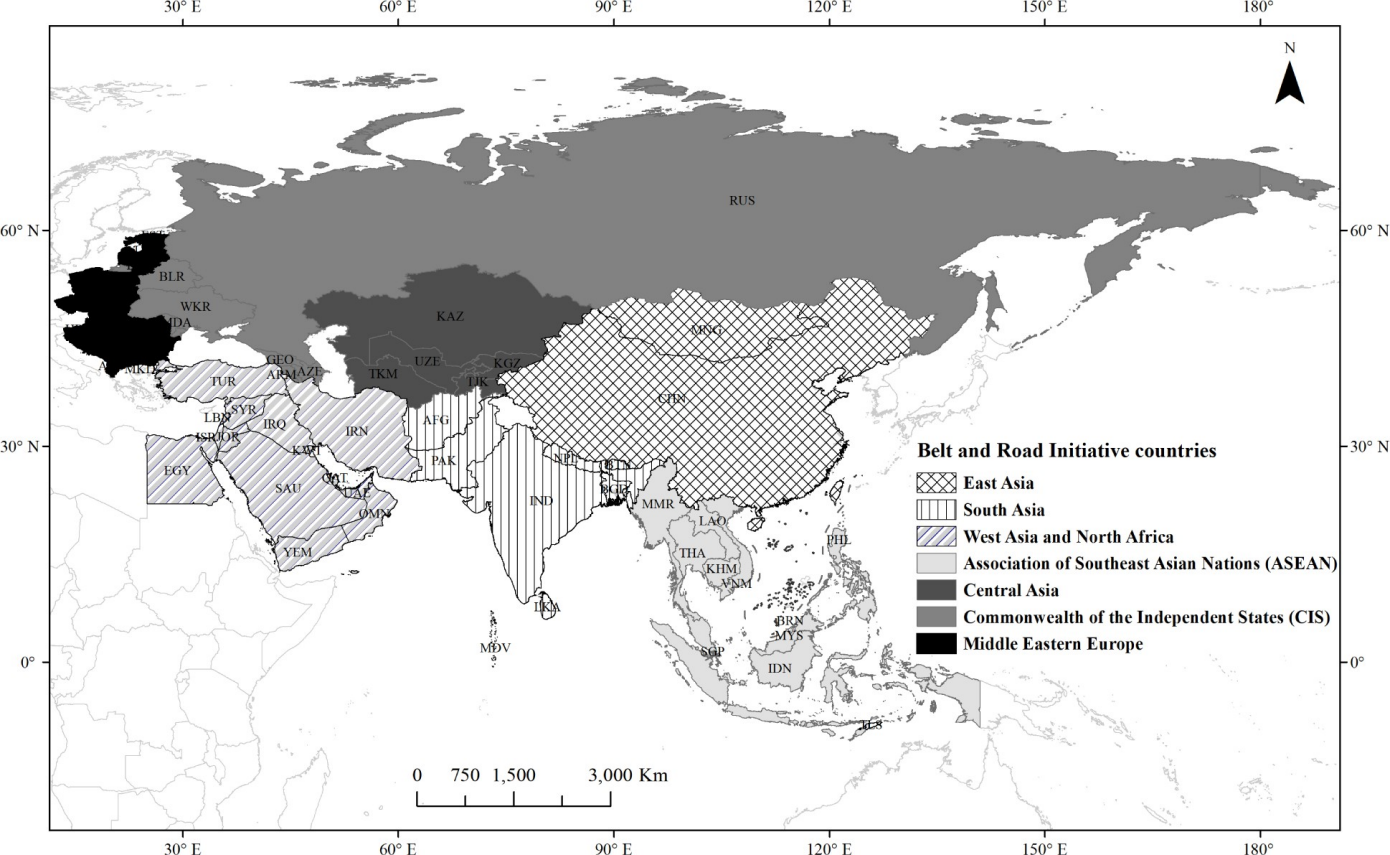
Geographic location of the BRI countries.

The BRI is an international and regional economic cooperation initiative proposed by China to further promote the development of economic globalization, aiming to build an open and inclusive network of regional interconnectivity and economic cooperation with no completely closed space [3]. According to data from the Belt and Road Portal, China has signed cooperation agreements with 64 BRI countries by December 2018. Based on the data and related research results [27, 28], we select China and other 64 BRI countries as the study area. The 65 countries have a total population of 4.4 billion, accounting for 64% of the world’s total, and an economic aggregate of USD 21 trillion, accounting for about 30% of the world’s total GDP [25]. The 65 countries can be divided into 7 subregions, namely East Asia, Association of Southeast Asian Nations (ASEAN), South Asia, West Asia and North Africa, Central Asia, Middle Eastern Europe and Commonwealth of the Independent States (CIS) (Fig 1).

### 2.2 Evaluation index

CESV refers to an essential attribute with which the economic system is easily damaged due to its sensitivity to various internal and external disturbances and its lack of ability to respond to adverse disturbances. To assess the CESV more comprehensively, we consider the domestic economic system, external economic system and financial system and construct an indicator system of CESV for the BRI countries (Table 1). This is constructed from the concept of the comprehensive vulnerability, considering the scientificity and feasibility of the indicator system, the availability and representativeness of the index data, and the previous research basis on economic vulnerability [6, 29]. For example, Ren et al. selected indicators such as inflation rate, exchange rate and proportion of actual utilized foreign capital in GDP [20]. Li et al. selected per capita GDP, total import and export volume, fixed asset investment and other indicators [30]. Hu and Wang selected indicators such as per capita GDP, GDP growth rate, inflation rate, the ratio of current account balance to GDP, currency and price stability, and GDP growth volatility in recent ten years to assess the vulnerability of the economic system [23]. In this study, the CESV is taken as the target layer, and the DESV, the EESV and FSV are taken as the sub-target layers, which contain 8 criteria layers and 26 evaluation indicators.

**Table 1:** Economic system vulnerability evaluation index system for the BRI countries.

Target layer	Sub-target layer	Criteria layer	Indicators	Units	Weights
CESV (A)	DESV (B1)	Macroeconomic development vulnerability (C1)	Per capita GDP (D1)	$	0.01
			Consumption increase rate (D2)	%	0.06
			Ratio of gross fixed capital formation to GDP (D3)	%	0.04
		Industrial development vulnerability (C2)	Industrialization level (Ratio of secondary industrial added value to GDP, D4)	%	0.04
			Service industry level (Ratio of tertiary industrial added value to GDP, D5)	%	0.08
		Macroeconomic operation vulnerability (C3)	GDP growth rate (D6)	%	0.24
			Inflation rate (D7)	%	0.04
			Saving ratio (D8)	%	0.14
			Unemployment rate (D9)	%	0.04
	EESV (B2)	Foreign trade system vulnerability (C4)	Ratio of trade volume to GNP (D10)	%	0.04
			Ration of external balance on goods and services to GDP (D11)	%	0.02
			Export growth rate (D12)	%	0.01
			Import growth rate (D13)	%	0.002
		External capital system vulnerability (C5)	Growth rate of foreign investment in actual use (D14)	%	0.003
			Change rate of exchange rate (D15)	%	0.003
			Debt ratio (D16)	%	0.03
	FSV (B3)	Financial monitoring system vulnerability (C6)	Ratio of the money supply to GDP (D17)	%	0.02
			Growth rate of broad money supply (D18)	%	0.03
			Real deposit interest rate (D19)	%	0.002
			Loan rate (D20)	%	0.04
		Banking system vulnerability (C7)	Ratio of credit scale to GDP (D21)	%	0.01
			Ratio of bank current reserves to assets (D22)	%	0.01
			Ratio of non-performing loans to total loans (D23)	%	0.03
			Ratio of bank capital to assets (D24)	%	0.04
		Stock market vulnerability(C8)	Ratio of stock value to GDP (D25)	%	0.03
			Stock index volatility (D26)	%	0.02

Specifically, the DESV is composed of macroeconomic development vulnerability, industrial development vulnerability and macroeconomic operation vulnerability, involving 9 indicators. Macroeconomic development vulnerability involves 3 indicators of per capita GDP (D1), consumption increase rate (D2) and ratio of gross fixed capital formation to GDP (D3); industrial development vulnerability involves 2 indicators of industrialization level (D4) and service industry level (D5). In addition, macroeconomic operation vulnerability involves 4 indicators of GDP growth rate (D6), inflation rate (D7), saving ratio (D8) and unemployment rate (D9). EESV involves 7 indicators, where foreign trade system vulnerability reflects the exchange of goods and services based on the development of productivity. This is made up of 4 indicators which are ratio of trade volume to GNP (D10), ratio of external balance on goods and services to GDP (D11), export growth rate (D12), and import growth rate (D13). Also, external capital system vulnerability reflects the economic relationship between a country and other countries and regions, involving 3 indicators of the growth rate of foreign investment in actual use (D14), the change rate of exchange rate (D15) and debt ratio (D16). FSV involves 10 indicators, where financial monitoring system vulnerability reflects the ability of steady operation and development of financial institutions, involving 4 indicators of the ratio of the money supply to GDP (D17), the growth rate of broad money supply (D18), real deposit interest rate (D19) and loan rate (D20). Furthermore, banking system vulnerability reflects financial risks and the healthy development of the financial industry. This is made up of 4 indicators which are the ratio of credit scale to GDP (D21), the ratio of bank current reserves to assets (D22), the ratio of non-performing loans to total loans (D23), and the ratio of bank capital to assets (D24). Stock market vulnerability reflects the development of the stock market, involving 2 indicators of ratio of stock value to GDP (D25) and stock index volatility (D26). In this study, the CESV of 65 BRI countries is calculated based on the panel data of 20 years (2000–2019). The assessment indicators in this study are all selected from the World Bank Database.

### 2.3 Methods

#### 2.3.1 Vulnerability calculation method

The vulnerability of economic system is calculated according to the “sensitivity-adaptability” function [26]. The vulnerability calculation formula is shown as below:

V=S−A (1)

where *V*, *S* and *A* is the vulnerability, sensitivity and adaptability of the system. The vulnerability depends on the sensitivity and adaptability of the system. The sensitivity represents the response degree of a system to external disturbance. The adaptability is the ability of the system to maintain and recover its structure in the face of external disturbance. Taking per capita GDP, an index of macroeconomic system, as an example, its sensitivity is expressed as inter-annual fluctuations of per capita GDP from 2000 to 2019. The formula for sensitivity calculation is as follows:

$$S_{j}=\frac{\sum_{i=1}^{n}\left|F_{i}-\bar{F}\right|}{\bar{F}}$$ (2)

where *F*_*i*_ is the value of index *j* in the year *i*; $\bar{F}$ is the average value of index *j* from 2000 to 2019. *S*_*J*_ (Sensitivity) is the variable rate of index *j*, which reflects the degree of dispersion of the average value of index *j* within the relatively specific time from 2000 to 2019.

Adaptation of a system can be defined as a measure of maintenance of the system in a relatively stable state. Thus, the trend of variability of a system is used to measure its deviation from the stable state, and referred to as the adaptability of the system. If the change trend of variability increased, it suggests an unstable system to adapt to external disturbance, and may indicate increasing vulnerability. Over a specific period, the adaptability can be expressed by the slope of the linear fitting trend line of inter-annual variability of the economic system index. For the per capita GDP index, the adaptability of the sub macroeconomic system was defined by the slope of the linear fitting trend line for inter-annual variability of per capita GDP from 2000 to 2019. The slope can be calculated by the least square fitting function as follows:

y=Ax+B (3)

$$A_{j}=\frac{n\sum xy_{j}-(\sum x)(\sum y_{j})}{n\sum x^{2}-{\left(\sum x\right)}^{2}}$$ (4)

where *A*_*j*_ is the changing trend of the variability of index *j*, namely the adaptability of index *j*, *x* refers to the time serial numbers, corresponding to the year from 2000 to 2019, and B is the intercept. *y*_*j*_ is the objective variable of index *j*, which is the value calculated through subtracting the value of indicator *j* from the average value of indicator *j* from 2000 to 2019. In addition, the sensitivity and adaptability calculated according to the previous formula may not be in the same dimension. In order to analyze the regional differences in vulnerability, the calculation results of sensitivity and adaptability need to be standardized separately before calculating the vulnerability.

#### 2.3.2 Entropy weight method

The entropy weight method (EWM) is an objective evaluation method. It is more credible than the weight determined by the subjective weighting method [31]. Its advantage is that it can avoid the interference of human factors and improve the objectivity of the comprehensive evaluation result. At present, the EWM has been widely used in engineering technology, social economy and other fields [32]. The EWM is mainly used to calculate the entropy weight of each indicator with the information entropy based on the degree of variation, and then correct the weight of each indicator by entropy weight, to obtain a more objective indicator weight. Generally, the smaller entropy weight of an indicator determined by the information entropy index weight determination method indicates a greater degree of variation and more information, thus playing a greater role in comprehensive assessment and having a greater weight [33]. Therefore, this study reflects the weight of each indicator of the vulnerability of a comprehensive economic system by the entropy weight method, which is highly applicable.

The first step in EWM is standardization [34].The positive and negative standardized formulas are as follows:

$$Positive\;index\;calculation:\;x_{ij}^{\prime }=\frac{x_{ij}-min(x_{j})}{max(x_{j})-min(x_{j})}$$ (5)

$${Negative\;index\;calculation:\;x}_{ij}^{\prime }=\frac{max(x_{j})-x_{ij}}{max(x_{j})-min(x_{j})}$$ (6)

Afterward, *y*_*ij*_ is generated by

$$y_{ij}=\frac{x_{ij}^{\prime }}{\sum_{i=1}^{n}x_{ij}}$$ (7)

In EWM, the entropy value *e*_*j*_ of the *j* th is defined as

$$e_{j}=-\frac{\sum_{i=1}^{n}y_{ij}ln(y_{ij})}{ln(n)}$$ (8)

when *y*_*ij*_ = 0, *y*_*ij*_×*lny*_*ij*_ is defined to be 0.

*E*_*j*_ lies in the [0,1] domain. In EWM, the weight parameter *w*_*j*_ is calculated as

$$w_{j}=\frac{1-e_{j}}{\sum_{j=1}^{m}1-e_{j}}$$ (9)

Comprehensive score calculation is as follows:

$$s_{i}=\sum_{j=1}^{m}w_{j}y_{ij}$$ (10)

#### 2.3.3 Obstacle factor diagnosis method

On the basis of the assessment of economic system vulnerability, not only the current vulnerability is measured, but also the main obstacle factors that affect the level of economic system vulnerability should be identified. The obstacle degree model is often used to diagnose the obstacle factors affecting the development of things based on the comprehensive evaluation mode [35]. Therefore, this paper introduces the obstacle degree model to carry on the pathological diagnosis to the influencing factors of the economic system vulnerability. The obstacle degree model mainly investigates the impact of sub-target layer B, criterion layer C and indicator layer D on the differences in the distribution of CESV in different countries, and provides path guidance for vulnerability mitigation. The obstacle degree model analyzes and diagnoses by using three indicators: contribution degree, index deviation degree and obstacle degree. The specific calculation formula is as follows [36]:

Factor contribution *E*_*i*_ in the obstacle degree model [37] is the contribution of a single factor to the overall goal:

$$F_{i}=W_{i}\times R_{ik}$$ (11)

where, *W*_*i*_ is the weight of the criterion level indicator *i*; *R*_*ik*_ is the weight of the single indicator *k* among the criterion level indicators.

Indicator deviation *I*_*ij*_ is the difference between single-factor indicators and system development goal, where *I*_*ij*_ = 1−*x*_*ij*_, indicating the difference between the standardized value of a single indicator and 1. Obstacle degree *O*_*ij*_ is the impact of a single indicator or criterion layer on the vulnerability of a comprehensive economic system:

$$O_{ij}=\frac{F_{i}I_{ij}}{\sum_{i=1}^{m}F_{i}I_{ij}}$$ (12)

## 3. Results

### 3.1 Characteristics of CESV of the BRI countries

#### 3.1.1 Spatial pattern of CESV of the BRI countries

**Fig 2:**
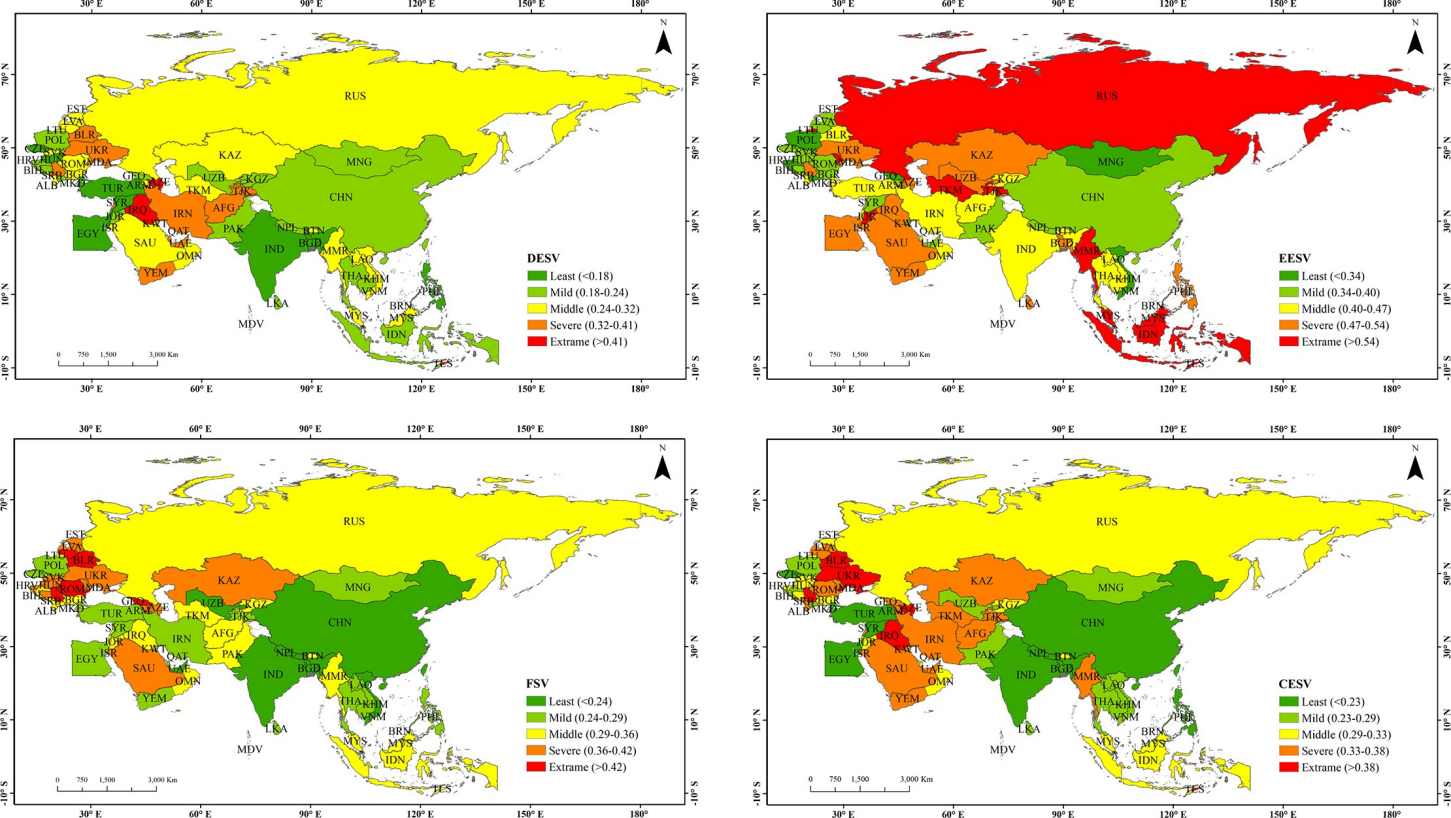
Spatial patterns of economic system vulnerability of the BRI countries (a. DESV, b. EESV, c. FSV, and d. CESV).

Through calculating and analyzing all the indicators and data, we have obtained the DESV, external economic system, financial system and comprehensive economic system of the BRI countries. The classification of vulnerability assessment results according to the natural breaking point method is shown in Fig 2. The DESV of the countries in central and southern West Asia, eastern Central and Eastern Europe and northern Central Asia is higher than that of the countries in East Asia, South Asia and ASEAN (Fig 2A). The extremely vulnerable countries mainly include Azerbaijan and Iraq in West Asia. The severely vulnerable countries are Iran, Yemen, Afghanistan, Tajikistan, the United Arab Emirates, Belarus and Ukraine. Russia in CIS, Kazakhstan and Turkmenistan in Central Asia, Saudi Arabia in West Asia, Myanmar and Laos in ASEAN are the concentrated distribution areas of moderately vulnerable countries. Countries in other regions, such as East Asia, South Asia and eastern West Asia, are basically at the level of mild or mild vulnerability. In countries with higher vulnerability, the high DESV is mainly caused by unbalanced industrial development, unstable macroeconomic operation, poor economic foundation, and low industrialization level.

The EESV of the BRI countries are generally high, showing a pattern of higher vulnerability in West Asia, Central Asia and ASEAN than that in East Asia and South Asia (Fig 2B). The extremely vulnerable countries include Indonesia, Russia, Turkmenistan and Myanmar. The severely vulnerable countries include Tajikistan, Uzbekistan, Saudi Arabia, Egypt, Yemen and Ukraine. The moderately vulnerable countries are mainly concentrated in Iran, Afghanistan, Turkey and Oman in West Asia, India in South Asia and Thailand, Laos and Cambodia in ASEAN. Mild vulnerability areas are mainly distributed in central and Eastern Europe and East Asia regions. The FSV of the BRI countries is generally at a moderate level, showing a pattern of higher vulnerability in West Asia and Central and Eastern Europe than that in East Asia and South Asia (Fig 2C). The distribution of extremely vulnerable countries is concentrated, mainly in central and Eastern Europe. The severely vulnerable countries are scattered, mainly including Kazakhstan, Saudi Arabia, Ukraine, Azerbaijan, Hungary and so on. Mild vulnerability areas are mainly distributed in South Asia, East Asia, east Asia and some ASEAN countries. In countries with a relatively high vulnerability of the financial system, the situation is mainly caused by the imperfect financial system and capital market, and the rigid regulation of the indicators such as interest rates, currencies, and stocks.

The spatial distribution of CESV of the BRI countries shows that there are significant variations in the vulnerability of a comprehensive economic system (Fig 2D). The CESV of East Asia, South Asia and ASEAN countries is lower than that of the countries in Central and Eastern Europe, Central Asia and West Asia. The extremely vulnerable countries are mainly concentrated in the eastern part of Central and Eastern Europe, including Belarus, Ukraine, Azerbaijan and Iraq. The severely vulnerable countries are mainly concentrated in West Asia, including Iran, Saudi Arabia, Oman and Afghanistan, as well as Latvia and Romania in central and Eastern Europe, Kazakhstan and Turkmenistan in Central Asia and Myanmar in ASEAN. The 16 countries including Russia, Indonesia, Malaysia, Oman, Slovakia, and Bulgaria are moderately vulnerable. Eastern and South Asia regions such as China, Mongolia, India, Laos, Vietnam, and parts of Eastern Europe and Western Asia such as Egypt and Poland are mildly vulnerable. The economic systems of most countries of ASEAN, East Asia and South Asia are the least susceptible to the impact, and they are low in terms of CESV.

In general, it can be seen from Fig 2 that the DESV, the FSV and the CESV are similar in spatial distribution. The regions with moderate and above vulnerability are all located in Central and Western Asia and Russia of the CIS, while those with mild vulnerability are concentrated in East Asia and South Asia. The EESV is quite different from the CESV in spatial distribution. Except for mild vulnerability in parts of East Asia, Central and Eastern Europe and parts of South Asia, other regions are moderately, severely and extremely vulnerable. In terms of the reasons, the CESV of Asia countries is mainly caused by high economic losses and economic instability due to poor economic foundations (low GDP), industrialization level (incomplete industrial system), and underdeveloped financial markets. Secondly, due to the unstable macroeconomic operation, they are susceptible to economic fluctuations of macro-structural indicators such as inflation and savings. The small economic aggregate and single industrial structure may make their economic development affected by external risks. In addition, the lagged development of the financial market, the imperfect capital market, and the rigid regulation of interest rates, currencies, stocks may severely hinder the free flow of funds for inter-regional development, thus leading to a greater negative impact on the comprehensive economic system.

**Fig 3:**
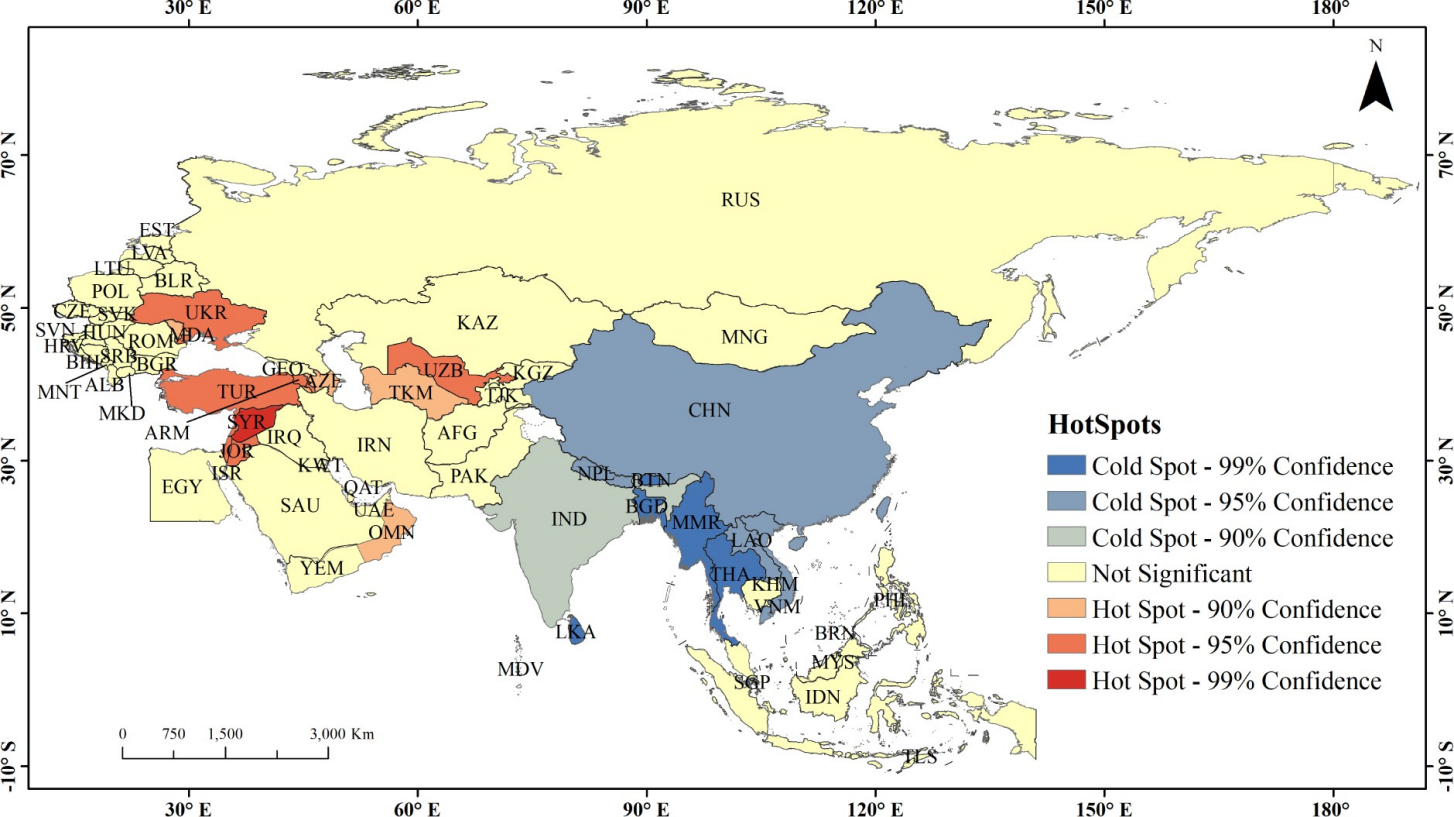
Hotspot analysis of CESV of the BRI countries.

In this study, the spatial agglomeration of the CESV of the BRI countries is further analyzed by global spatial autocorrelation [38] and local hot spot analysis (Fig 3). The results indicate that the CESV of the BRI countries is agglomerated, with Moran’s I of 0.05 under the Confidence level of 5%. It presents a pattern with hot spots in eastern and southern West Asia and cold spots in East Asia, northern ASEAN and some parts of South Asia. The hot spots are mainly distributed in Turkey, Syria and Jordan in west Asia and Uzbekistan and Turkmenistan in Central Asia, showing an obvious spatial high-value aggregation status. While, the cold spots are mainly distributed in China, India, Burma, Vietnam, Laos, etc., showing an obvious spatial low-value aggregation status. As indicated above, the distribution pattern of cold and hot spots is the same as the spatial distribution pattern of the CESV, namely, high in Central Asia and West Asia, and low in South Asia and East Asia.

#### 3.1.2 Differences in CESV of the BRI countries

**Fig 4:**
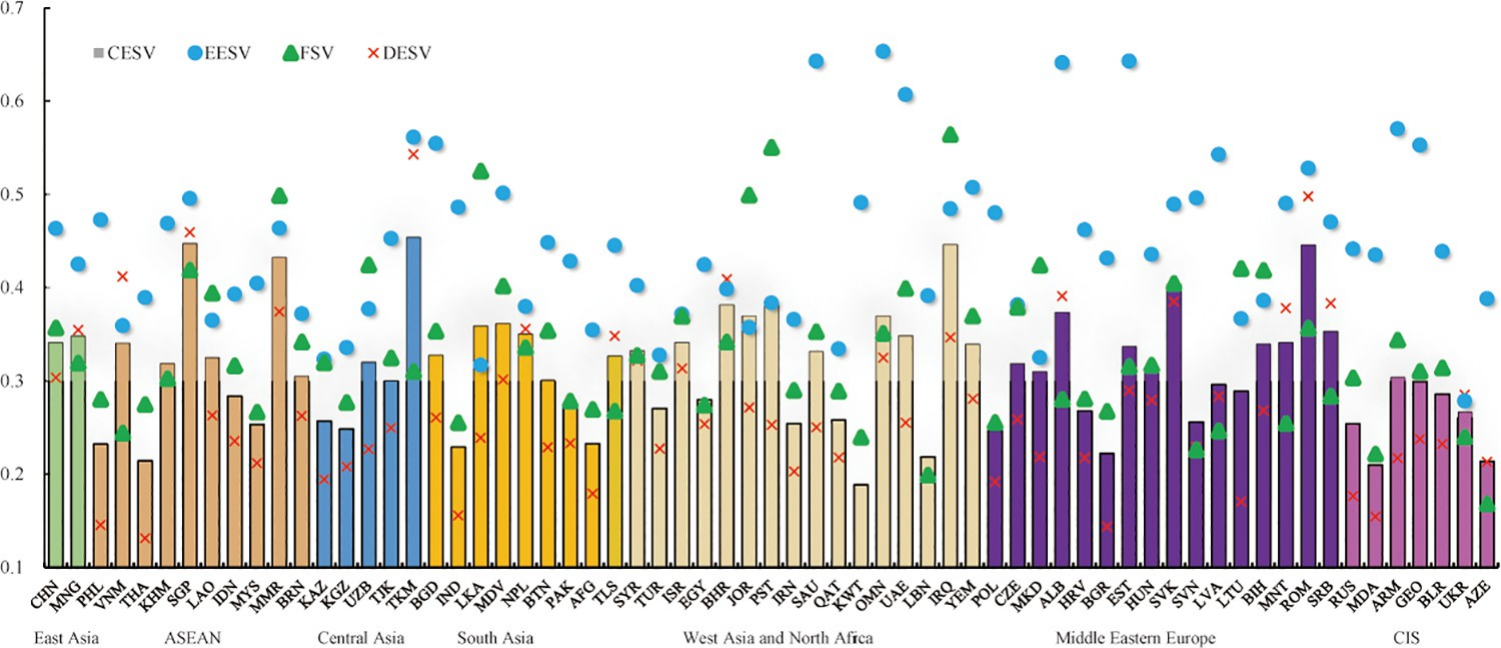
Values of EESV, FSV, DESV and CESV of each BRI country.

The DESV is 0.08–0.54, with an average value of 0.27; the EESV is 0.28–0.65, with an average value of 0.44; and the FSV is 0.17–0.56, with an average value of 0.33. The DESV is low, followed by the FSV. While the EESV is higher than that of the other two systems of the BRI countries. The CESV of the BRI countries indicator is 0.19–0.45, with an average value of 0.31, which is the moderate vulnerability. In addition, Fig 4 also shows that there is a clear gap in the CESV between different countries, with the highest score can be over twice as much as the lowest score. The grouping statistics of the vulnerability indicate that the average vulnerability indicator of highly vulnerable countries can be double of that of lowly vulnerable countries, with an obvious difference between the groups.

**Fig 5:**
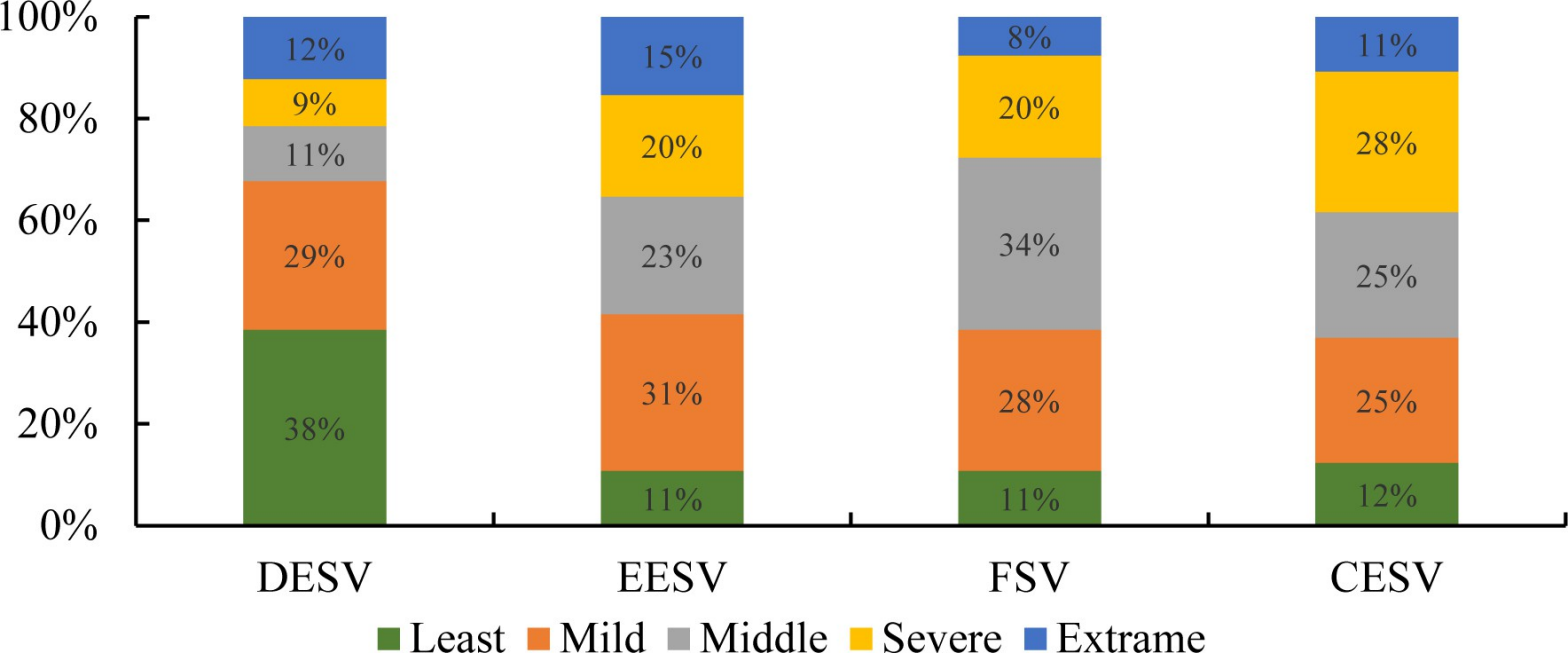
Hierarchical statistics of the vulnerability of the BRI countries.

Further statistical analysis of the vulnerability classification results of the sub-target layer indicates that, the countries with DESV distributed in the top two levels of vulnerability accounts for above 67%, and most countries are mild in terms of vulnerability (Fig 5). The distribution of the EESV and the FSV are similar, with the proportion of countries distributed in the top two levels reaching more than 39%. The number of moderately vulnerable and severely vulnerable countries is equal, accounting for 11% and 20% of the total respectively. Slightly vulnerable countries are roughly equally distributed, accounting for 31% and 28% of the total respectively. In addition, moderately vulnerable states account for 23% and 34% of the total number of countries, respectively. From the perspective of the distribution of CESV, the number of slightly vulnerable countries is the same as that of the moderately vulnerable countries (16 countries), accounting for 25%. The severely vulnerable countries account for 28% of the total, while the extremely vulnerable and moderately vulnerable countries account for 11% and 12% of the total, respectively. The distribution of CESV of the BRI countries presents a pattern of more in the middle and less at both ends. All countries have their economic problems in the process of rapid economic development and have varying degrees of vulnerability, but the overall vulnerability of a comprehensive economic system is moderate.

### 3.2 Influencing factors of CESV of the BRI countries

The study further analyzes and explores the obstacles affecting the CESV of the BRI countries based on the obstacle degree model. The obstacles of the indicator layer are ordered according to the degree of the obstacles, and only the top five ones are listed. As shown in overall obstacle statistics (Table 2), among the vulnerability assessment indicators, the obstacles affecting the CESV of the BRI countries mainly include GDP growth rate (D6), saving ratio (D8), and ratio of bank capital to assets (D24). The top 3 indicators affecting the CESV of different countries appear frequently, the frequencies were 62, which indicating that the obstacle factors are unevenly distributed in various subsystems, and are mainly concentrated on macroeconomic operations and banking system. It also suggests that the countries should continue to improve the GDP growth rate, the savings rate and bank capital ratios, this is to keep the macroeconomic operation stable and optimize the banking system, so as to reduce the vulnerability of the economic system. The following obstacles are service industry level (D5), industrialization level (D4) and loan rate (D20), with the frequency of 47, 40 and 18, indicating that the BRI countries should also focus on industrial development and financial monitoring system. Therefore, given the impact of these indicators on reducing vulnerability, the BRI countries should focus on macroeconomic operations (improving GDP growth and saving ratio), simulate the economic and market dynamism, promote industrial development, especially the service and manufacturing industries, and optimize the economic structure, banking system and financial system in the future development, which is key to maintain the stability of the economic system and alleviate the vulnerability of the BRI countries.

**Table 2:** Obstacle factor and obstacle degree of integrated economic system of the BRI countries.

Countries	First obstacle factor	O_*ij*_	Second obstacle factor	O_*ij*_	Third obstacle factor	O_*ij*_	Fourth obstacle factor	O_*ij*_	Fifth obstacle factor	O_*ij*_
ALB	D24	0.10	D6	0.09	D8	0.09	D4	0.07	D5	0.07
AFG	D20	0.11	D6	0.10	D24	0.10	D8	0.09	D9	0.08
EGY	D6	0.09	D20	0.09	D24	0.09	D8	0.08	D4	0.07
UAE	D24	0.10	D6	0.10	D20	0.09	D8	0.09	D23	0.07
SYR	D6	0.09	D8	0.09	D24	0.08	D20	0.08	D4	0.07
OMN	D6	0.10	D24	0.10	D20	0.08	D8	0.08	D9	0.06
AZE	D8	0.12	D20	0.12	D24	0.08	D4	0.07	D5	0.07
EST	D8	0.11	D6	0.09	D24	0.09	D4	0.08	D5	0.08
PAK	D6	0.10	D24	0.09	D8	0.09	D20	0.09	D4	0.07
BHR	D8	0.09	D6	0.09	D24	0.09	D20	0.08	D4	0.07
BLR	D8	0.12	D24	0.12	D6	0.09	D4	0.08	D5	0.08
BGR	D8	0.10	D24	0.10	D6	0.09	D20	0.08	D4	0.08
MKD	D6	0.10	D8	0.10	D24	0.09	D20	0.08	D4	0.07
POL	D6	0.10	D8	0.09	D24	0.09	D4	0.08	D5	0.08
BIH	D8	0.10	D6	0.09	D4	0.08	D5	0.08	D20	0.08
BTN	D20	0.10	D6	0.09	D8	0.09	D24	0.08	D4	0.08
TLS	D6	0.14	D20	0.13	D24	0.12	D9	0.09	D23	0.08
RUS	D8	0.10	D24	0.09	D6	0.08	D4	0.08	D5	0.08
PHL	D6	0.10	D8	0.09	D24	0.09	D20	0.07	D4	0.06
GEO	D6	0.10	D8	0.10	D20	0.08	D9	0.07	D4	0.07
KAZ	D8	0.11	D24	0.10	D20	0.09	D6	0.08	D4	0.07
MNT	D6	0.11	D24	0.10	D20	0.10	D8	0.09	D25	0.06
KGZ	D6	0.10	D8	0.10	D24	0.09	D4	0.06	D5	0.06
KHM	D8	0.10	D6	0.09	D20	0.08	D24	0.08	D4	0.07
CZE	D6	0.09	D8	0.09	D24	0.08	D4	0.08	D5	0.08
QAT	D8	0.10	D24	0.10	D20	0.09	D6	0.07	D4	0.07
KWT	D24	0.10	D8	0.10	D20	0.08	D6	0.08	D4	0.07
HRV	D8	0.10	D24	0.09	D6	0.09	D20	0.09	D4	0.06
LVA	D8	0.11	D24	0.10	D6	0.09	D4	0.07	D5	0.07
LAO	D6	0.10	D20	0.09	D8	0.09	D24	0.09	D4	0.07
LBN	D24	0.11	D6	0.10	D20	0.08	D23	0.07	D18	0.07
LTU	D8	0.11	D6	0.10	D24	0.08	D4	0.08	D5	0.08
ROM	D6	0.11	D8	0.11	D24	0.10	D4	0.07	D5	0.07
MDV	D6	0.10	D20	0.09	D8	0.09	D24	0.09	D3	0.06
MYS	D6	0.11	D24	0.10	D8	0.10	D20	0.09	D9	0.07
MNG	D6	0.10	D24	0.09	D8	0.09	D4	0.07	D5	0.07
BGD	D6	0.10	D8	0.09	D24	0.08	D20	0.08	D4	0.07
MMR	D8	0.11	D24	0.10	D20	0.10	D6	0.08	D9	0.07
MDA	D6	0.10	D8	0.10	D24	0.10	D4	0.08	D5	0.08
NPL	D6	0.10	D8	0.10	D24	0.09	D20	0.08	D23	0.07
SRB	D8	0.13	D24	0.11	D6	0.11	D9	0.07	D3	0.07
SAU	D6	0.10	D24	0.10	D8	0.10	D20	0.09	D9	0.08
LKA	D6	0.10	D8	0.09	D24	0.09	D20	0.08	D4	0.07
SVK	D8	0.10	D6	0.10	D24	0.10	D4	0.08	D5	0.08
SVN	D6	0.10	D8	0.10	D24	0.09	D4	0.07	D5	0.07
TJK	D20	0.12	D6	0.11	D8	0.10	D24	0.10	D23	0.08
THA	D8	0.10	D6	0.09	D20	0.08	D24	0.08	D4	0.07
TUR	D6	0.09	D8	0.09	D24	0.08	D20	0.08	D4	0.07
TKM	D6	0.12	D8	0.10	D24	0.09	D20	0.09	D4	0.08
BRN	D8	0.11	D20	0.10	D6	0.09	D24	0.09	D9	0.07
UKR	D8	0.10	D24	0.09	D6	0.08	D20	0.08	D7	0.08
UZB	D6	0.10	D8	0.09	D20	0.09	D24	0.09	D18	0.06
SGP	D8	0.10	D20	0.10	D24	0.10	D6	0.09	D23	0.06
HUN	D6	0.10	D8	0.10	D24	0.09	D4	0.08	D5	0.08
ARM	D8	0.10	D4	0.09	D5	0.09	D6	0.08	D9	0.08
YEM	D20	0.11	D24	0.10	D8	0.09	D9	0.08	D18	0.07
IRQ	D6	0.14	D24	0.12	D20	0.12	D8	0.11	D9	0.09
IRN	D24	0.10	D8	0.09	D6	0.09	D9	0.07	D20	0.07
ISR	D6	0.10	D8	0.09	D20	0.09	D24	0.08	D4	0.07
IND	D6	0.09	D24	0.09	D8	0.09	D20	0.08	D9	0.06
IDN	D6	0.11	D8	0.10	D24	0.08	D20	0.08	D3	0.07
JOR	D24	0.10	D20	0.09	D6	0.09	D8	0.08	D4	0.08
PST	D6	0.11	D20	0.09	D24	0.09	D8	0.08	D9	0.07
VNM	D6	0.10	D24	0.08	D8	0.08	D20	0.08	D23	0.06
CHN	D16	0.03	D24	0.09	D22	0.02	D23	0.06	D21	0.02

## 4. Conclusions and discussion

### 4.1 Conclusions

This study constructs a vulnerability assessment indicator system for comprehensive economic systems of the BRI Countries from three dimensions of the domestic economic system, external economic system and financial systems, and the analysis of the main factors affecting the vulnerability is presented based on the obstacle degree model. In the context of the BRI, the systematic assessment of the development status of economic systems in the BRI countries, and the identification of regional CESV is of great importance to the achieving of the stability and sustainable development of the BRI.

The CESV of the BRI countries presents a pattern of lower vulnerability in East Asia, South Asia and ASEAN countries than that in the Central and Eastern Europe, Central Asia and West Asia countries. This is similar to the DESV and FSV, but different to the EESV. The spatial distribution of DESV shows that the overall vulnerability of central and southern West Asia, eastern Central and Eastern Europe and northern Central Asia is higher than that of East Asia, South Asia and ASEAN. The most vulnerable countries are mainly in Western Asia, including Azerbaijan and Iraq. The high DESV is mainly caused by unbalanced industrial development, unstable macroeconomic operation, poor economic foundation, and low industrialization level. The overall spatial distribution of EESV is high, showing a pattern of higher vulnerability in west Asia, Central Asia and ASEAN than that in East Asia and South Asia. The most vulnerable countries are Indonesia, Russia, Turkmenistan and Myanmar. The overall spatial distribution of FSV is at a moderate level, showing a pattern of higher vulnerability in west Asia and Central and Eastern Europe than that in East Asia and South Asia. However, the overall spatial distribution of FSV is at a moderate level, showing a pattern of higher vulnerability in west Asia and Central and Eastern Europe than that in East Asia and South Asia. The extremely vulnerable countries are mainly concentrated in Central and Eastern Europe. The higher FSV is generally caused by the imperfect financial system and capital market, and the rigid regulation of the indicators such as interest rates, currencies, and stocks. The distribution of CESV of the BRI countries is concentrated, presenting a pattern with hot spots in eastern and southern West Asia and cold spots in East Asia, northern ASEAN and some parts of South Asia. The identification of impact factors based on the obstacle degree model indicates that the main obstacles to the CESV of the BRI countries include GDP growth rate (D6), saving ratio (D8), ratio of bank capital to assets (D24), service industry level (D5), industrialization level (D4) and loan rate (D20). Therefore, the BRI countries can further improve macroeconomic operations, stimulate the economy and market dynamism, and take measures to promote industrial development, especially the development of the service industry and manufacturing industry, and optimize the economic structure, banking system and financial system.

### 4.2 Discussion

CESV is an important indicator for measuring the health and stability of the development of the economic system in a region or country, and also a research hotspot at present. In the existing studies, there are only a few studies on the assessment of CESV of the BRI countries. Therefore, this study has certain research value and significance. In this study, based on the concept of integrated vulnerability, CESV is taken as the target layer, DESV, EESV and FSV as the sub-target layer, and entropy weight method is used to assess the vulnerability of the economic system in BRI countries. In addition, we analyzed the spatial agglomeration pattern of CESV in the BRI countries, and analyzed the main factors that influence vulnerability based on the obstacle degree model. The analyses can scientifically reflect the development of economic systems of the BRI countries. Based on which, we have proposed relevant measures, which will be of great significance for reducing the vulnerability of the comprehensive economic system and achieving sustainable development goals. However, there are still certain limitations in this paper. Firstly, given a large number of factors involved in economic vulnerability, it is hard to include the factors such as institutional, management and cultural into the indicator system. Secondly, the spatial scale can be further refined. Due to the high difficulty in collecting data, this study is only conducted at country level, but considering the different economic development stages, some indicators may be different within countries, which may make it fail to reflect the differences within countries. Thirdly, although this study analyzes the present characteristics of the CESV of the BRI countries, it will also be important to predict or simulate the future changes in CESV of the BRI countries based on scenario analysis. Overall, the study provides insight into the assessment of CESV, which is relevant to the sustainable development. In the future, it can be considered to further build a more solid and comprehensive vulnerability assessment indicator system, and carry out studies on the BRI countries combining the macro scale with more refined scale and based on scenario analysis, thus to provide more specific decision supporting information in line with local conditions policy makers and project planners to better optimize measures to manage economic risks in BRI areas.

## Supporting information

S1 Data (XLS)
